# Suppressed Retinal Degeneration in Aged Wild Type and APP*swe*/PS1ΔE9 Mice by Bone Marrow Transplantation

**DOI:** 10.1371/journal.pone.0064246

**Published:** 2013-06-04

**Authors:** Yue Yang, Christine Shiao, Jake Frederick Hemingway, Nikolas L. Jorstad, Bryan Richard Shalloway, Rubens Chang, C. Dirk Keene

**Affiliations:** Department of Pathology, University of Washington, Seattle, Washington, United States of America; Charité-Universitätsmedizin Berlin, Germany

## Abstract

Alzheimer's disease (AD) is an age-related condition characterized by accumulation of neurotoxic amyloid β peptides (Aβ) in brain and retina. Because bone marrow transplantation (BMT) results in decreased cerebral Aβ in experimental AD, we hypothesized that BMT would mitigate retinal neurotoxicity through decreased retinal Aβ. To test this, we performed BMT in APP*swe*/PS1ΔE9 double transgenic mice using green fluorescent protein expressing wild type (wt) mice as marrow donors. We first examined retinas from control, non-transplanted, aged AD mice and found a two-fold increase in microglia compared with wt mice, prominent inner retinal Aβ and paired helical filament-tau, and decreased retinal ganglion cell layer neurons. BMT resulted in near complete replacement of host retinal microglia with BMT-derived cells and normalized total AD retinal microglia to non-transplanted wt levels. Aβ and paired helical filament-tau were reduced (61.0% and 44.1% respectively) in BMT-recipient AD mice, which had 20.8% more retinal ganglion cell layer neurons than non-transplanted AD controls. Interestingly, aged wt BMT recipients also had significantly more neurons (25.4%) compared with non-transplanted aged wt controls. Quantitation of retinal ganglion cell layer neurons in young mice confirmed age-related retinal degeneration was mitigated by BMT. We found increased MHC class II expression in BMT-derived microglia and decreased oxidative damage in retinal ganglion cell layer neurons. Thus, BMT is neuroprotective in age-related as well as AD-related retinal degeneration, and may be a result of alterations in innate immune function and oxidative stress in BMT recipient mice.

## Introduction

Advanced age represents the strongest risk factor for Alzheimer's disease (AD) but is also associated with degenerative changes of brain and retina in the absence of clinical disease. Progressive visual deficits, such as reduced visual acuity and spatial contrast sensitivity, acquired color deficiencies, and decreased temporal resolving capacity, occur with advancing age even in the absence of specific disease [1]. Age-related neurodegeneration of retinal nerve fiber layer, ganglion cell layer and inner nuclear layer, as well as optic nerve axons, have all been described [2], [3], [4], [5], [6], and there is increased amyloid precursor protein deposition in retinal ganglion cell layer (RGCL) from aged patients without AD [7].

Pathologic changes of the visual pathways are well-characterized in AD as well. AD patients commonly experience altered spatial contrast sensitivity, susceptibility to visual masks, impaired ocular motility and abnormal pattern electroretinogram [8]. In addition to damage to central cortical visual pathways late in AD, recent studies have demonstrated retinal ganglion cell (RGC) degeneration [9], decreased thickness of the retinal nerve fiber layer [10], and optic nerve degeneration [11] that could account for some AD-related visual dysfunction. Amyloid β (Aβ) deposition, a neuropathological hallmark of AD in brain [12], [13], [14], [15], is present in AD retina and lens [16], [17] as well as other age-related diseases of retina such as glaucoma and age-related macular degeneration [16], [18], [19], [20]. Aβ peptides are pleiotropic neurotoxins which directly damage neurons as well as indirectly cause neuron damage through neurotoxic innate immune activation [14], [21], [22], [23] mediated by microglia [24]. Microglia are the primary innate immune effector cells in the central nervous system (CNS), and respond to toxic stimuli via multiple functions, including migration to the insult, phagocytosis of toxic molecules or debris, and elaboration of immunomodulatory molecules, such as cytokines and chemokines, as well as directly neurotoxic reactive oxygen species (ROS) [25], [26]. Microglia can be both beneficial and harmful, depending on the balance between neuroprotective and cytotoxic functions [27], [28], [29], but innate immune activation is altered in chronic neurodegenerative conditions to promote exaggerated microglial responses and a predominance of neurotoxic proinflammatory and oxidative microglial activity [30]. Thus, an important therapeutic challenge is balancing deleterious and beneficial aspects of microglial activation to promote a neuroprotective phenotype.

While there continues to be much debate about the extent of peripheral (blood derived) monocyte engraftment in the CNS in the absence of experimental manipulation or disease [31], numerous studies have confirmed that bone marrow transplantation (BMT) combined with preconditioning irradiation results in robust engraftment of donor microglia in brain [32], [33], [34], [35] and retina [36], [37]. Previous studies in brain demonstrated that BM-derived microglial cells reduced the brain Aβ plaque burden in experimental AD [34]. We recently expanded upon this work in brain by demonstrating that high dose cranial irradiation alone does not alter cerebral Aβ burden in APP*swe*-PS1ΔE9 mice, that BM-derived microglia engraftment is increased in APP*swe*-PS1ΔE9 mice compared with wild type (wt) controls, and that BM-derived microglia engraftment is necessary for reduced Aβ [32].

As in AD patients, APP*swe*-PS1ΔE9 mice exhibit neurotoxic Aβ peptide deposition in retina [22], [38]. Here, we tested the hypothesis that BMT would be neuroprotective in retina in experimental AD by mediating a reduction in Aβ peptide levels and Aβ neurotoxicity. First, we further characterized the retinal neuropathology of APP*swe*-PS1ΔE9 mice and then examined retinas of wt and APP*swe*-PS1ΔE9 mice that received BMT from allogeneic wt;GFP donors. We confirmed robust BM-derived microglia engraftment in retina and identified significant reductions in Aβ. We also identified significantly increased RGCL neurons in APP*swe*-PS1ΔE9 and wt mice that received BMT, which prompted us to test the hypothesis that BMT was neuroprotective in AD-related as well as age-related retinal neurodegeneration. We show that BMT mitigates RGCL neuron loss due to age and AD, and provide evidence that age and AD-related neuroprotection by BMT may be mediated through MHC class II expression and reduced oxidative stress.

## Materials and Methods

### Animals

**Figure 1:**
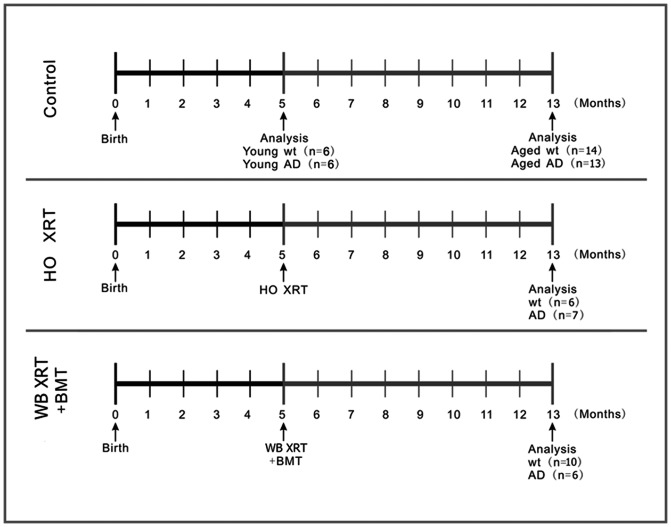
Experimental design. Control mice included wt and APP*swe*-PS1ΔE9 that received no irradiation or BMT and were euthanized at 5 and 13 months of age for analysis of retinal pathology. Additional control wt and APP*swe*-PS1ΔE9 mice received head only (HO) irradiation (XRT) at 5 months of age and were euthanized at 13 months of age for analysis of retinal pathology. Experimental groups included 5-month-old wt and APP*swe*-PS1ΔE9 mice that received lethal (10.5 Gy) whole body (WB) irradiation followed 24 hours later by retroorbital venous plexus injection of whole bone marrow from GFP-expressing wt mice and were then euthanized at 13 months of age for analysis of retinal pathology. Arrows mark the time points of generation and analysis of the control or treated mice.

The experimental groups and manipulations are depicted graphically in Fig. 1. All procedures in this study were approved by the University of Washington Institutional Animal Care and Use Committee. Mice were originally obtained from Jackson Laboratories (Bar Harbor, ME) and bred in our colony. BMT were performed in female B6C3F1/J hemizygous APP*swe*-PS1ΔE9 transgenic mice and their wt littermate controls using male C57BL/6 mice hemizygous for enhanced green fluorescent protein (GFP) mice as donors. The APP*swe* transgene encodes a mouse-human hybrid transgene containing the mouse sequence in the extracellular and intracellular regions and a human sequence within the Aβ domain with Swedish mutations K594N and M595L [39], [40]. The PS1ΔE9 transgene encodes exon-9-deleted human presenilin-1. Both transgenes are co-expressed under control of the mouse prion promoter with plaque deposition beginning at 5 months of age [39], [40]. GFP expression is under control of the β-actin promoter and cytomegalovirus enhancer.

### Irradiation and Bone Marrow Transplantation

Irradiation was applied to 5-month-old mice as a preconditioning therapy for BMT-recipients or to the head only as an irradiation control. 24 hours prior to BMT, APP*swe*-PS1ΔE9 double transgenic mice and their age-matched wt controls received whole body 10.5 Gy single dose irradiation at a rate of 2 Gy per minute from a Cesium-137 source (JL Shepherd, Model 81–14, San Fernando, CA). Control wt and APP*swe*-PS1ΔE9 mice were age-matched and either received no irradiation or had the same radiation dose applied to the head only with 5 cm of lead shielding (equivalent to 7.7 half-value layers  =  less than 0.5% of the total dose) to the neck, body and tail [32]. BMT followed an established protocol [32], [41]. BM cells were isolated from 10-week-old male donor GFP-expressing wt mice by flushing the femur and tibias with R10 media (RPMI with 10% fetal bovine serum). The samples were combined, passed through a 25-gauge needle filtered through a 70 µm nylon mesh, and centrifuged. Erythrocytes were lysed in ammonium chloride potassium (ACK) lysing buffer (Invitrogen, Carlsbad, CA) and the remaining leukocytes were resuspended in sterile phosphate buffered saline (PBS) at a concentration of ∼5×10^6^ viable nucleated cells per 200 µl. APP*swe*-PS1ΔE9 or wt BMT recipient females received retroorbital venous plexus injections one day after irradiation.

### Tissue Processing and Immunofluorescence

Eight months post-transplantation, recipient mice were given intraperitoneal (i.p.) injections of 50 µg/g body weight bromodeoxyuridine (BrdU) (Sigma-Aldrich, St. Louis, MO) for three consecutive days. 24 hours after the last BrdU administration, animals were anesthetized with avertin, and were transcardially perfused with ice-cold PBS. Eyes were rapidly dissected out and the cornea and lens were removed, and then were post-fixed in 4% PFA for 24 hours at 4°C and cryoprotected in 5%, 10% and 20% sucrose for 30 min each. Although whole mount retinas would provide better architectural cytomorphology, we chose to section the globes rather than perform whole mounts in order to preserve the ability to perform multiple immunostains in each eye. Eyes were oriented, embedded in Optimal Cutting Temperature (OCT) Compound (Tissue Tek, Torrance, CA), and were serially sectioned on a cryostat (Leica CM3050; Leica Microsystems GmbH, Wetzlar, Germany) at 25 µm or 40 µm thickness and were mounted on a repeating series of slides to ensure unbiased sampling. Sections were stored at −20°C until performing immunohistochemistry. Cryosections were blocked for 1 hour in 5% normal donkey serum (Jackson ImmunoResearch, West Grove, PA) containing 0.3% Triton X-100 in PBS and then were incubated with primary antibodies diluted in 0.3% Triton X-100 in PBS overnight at 4°C. For BrdU immunofluorescence, sections were pretreated in 2N HCl for 30 min at 37°C prior to blocking in 10% normal goat serum with 2% BSA (Sigma-Aldrich, St. Louis, MO). Primary antibodies included anti-ionized calcium binding adaptor molecule 1 (Iba-1) (Wako, Richmond, VA; 1∶500), anti-Aβ peptides (Covance, Princeton, NJ; 1∶1000), anti-NeuN (Millipore, Billerica, MA; 1∶500), anti-paired helical filament (PHF)-tau (Thermo Scientific, Rockford, IL; 1∶100), anti – 8 – hydroxydeoxyguanosine (8-OHdG) (Abcam, Cambridge, MA; 1∶200), anti-MHC class II (Novus Biologicals, Littleton, CO; 1∶400), and anti-BrdU (Accurate Chemical, Westbury, HY; 1∶400). The following day the sections were treated for three hours with species-appropriate secondary antibodies conjugated to Cy3 (Jackson ImmunoResearch, West Grove, PA; 1∶400) or Alexa Fluorescence 647 (Invitrogen, Carlsbad, CA; 1∶100). Coverslips were fixed using Prolong-Gold Anti-Fade with 4′, 6′-diaminido-2-phenylindole (DAPI) (Invitrogen, Carlsbad, CA) as a nuclear counterstain.

To determine the immunofluorescence relative intensity ( = per individual cell), two images of each retina (1 central and 1 peripheral) from different individuals per group were acquired through a fluorescent microscope (Melville, NY) at 200× magnification. All slides were previewed and optimum exposure time and gain was calculated for a standardized data acquisition. The MHC class II immunofluorescence relative intensity was evaluated on microglia along the RGCL, inner plexiform layer, inner nuclear layer, outer plexiform layer and outer nuclear layer. 8-OHdG intensity was measured on GCL neurons of all slides. A software-based processing with standardized background subtraction and analysis was applied to the digital images using Image J program (NIH, Bethesda, MD).

### Analysis of Engraftment of BM-derived Cells in Retina

To quantify retinal Iba-1-immunopositive microglia and BM-derived cells (GFP^+^), two nonadjacent central sections from both eyes of each mouse were analyzed at 200× magnification using a Nikon Eclipse 80i upright fluorescent microscope (Melville, NY) and StereoInvestigator software (MicroBrightfield, Williston, VT). The retina from each globe section was traced in StereoInvestigator. All Iba-1 immunopositive cells with a nucleus were counted throughout the total retinal area and cross-sectional thickness and recorded as Iba-1^+^, GFP^+^, or Iba-1/GFP-double positive. Cell density for the retina was determined by the total area of each cross-section divided by the total cell population. Cell density for retina layers was determined by the total length of each retina divided by the total cell population. Cells in the nerve fiber layer and RGCL, inner plexiform layer, inner nuclear layer, outer plexiform layer, and outer nuclear layer were specifically recorded as Iba-1^+^, GFP^+^, or Iba-1/GFP-double positive in relation to their specific layer. Non-irradiated, non-transplanted age matched wt and APP*swe*-PS1ΔE9 mice were used as controls. Cell counts were normalized to the total counting area from each section of retina. Observers were blinded to experimental conditions in all cases.

### Quantitation of Retinal Aβ and Paired Helical Filament (PHF)-tau

Aβ and PHF-tau quantitation was performed on representative retinal sections according to our previously published protocol [42] modified for fixed cryostat sections. Photomicrographs were acquired from standardized peripheral, middle, and central retina from each section using a Nikon Eclipse 80i upright fluorescent microscope (Mellville, NY) with the same magnification (200×), exposure time (200 ms), gain value (1), and other parameters for all the images. Optical sections were imported into the ImageJ software program (NIH, Bethesda, MD), converted to gray scale, and the total area of immunoreactivity was determined using a standardized histogram-based threshold technique. Total retinal area was used to normalize the data by computing the percent retinal area occupied by Aβ-immunoreactivity. For PHF-tau quantitation, a constant threshold value was applied to all the images using ImageJ and the number of pixels that matched or exceeded the set threshold value was calculated for each image and then divided by the total number of pixels in the image to determine the fluorescence intensity index. Results are expressed as a percent of wt control.

### Inner Retina Assessments

RGCL neurons were counted in sections on 20× fields. Only central sections located at or within 500 µm near the optic nerve head were used for counting. Within a section, NeuN^+^ cells were counted in a full length of retina on each side of the optic nerve head. This method was repeated on five sections per retina. For an individual retina the number was the average of the five counts. Six or more retinas of each genotype were quantified. Inner retinal atrophy was assessed by measuring inner retinal thickness using photomicrographs of preselected regions of central and peripheral retina in two non-adjacent central cross sections that were acquired at a magnification of 200× using a Nikon Eclipse 80i microscope. The thickness of the combined inner plexiform, ganglion cell, and nerve fiber layers (IPL-GCL-NFL) was measured using the ImageJ program. Apoptotic cell death was detected by a terminal deoxynucleotidyl transferase-mediated dUTP nick end labeling (TUNEL) assay (In Situ Cell Death Detection Kit, TMR red, Roche Ltd.) according to the protocol provided by the manufacturer. TUNEL and BrdU stained sections were double-labeled using NeuN antibody; BrdU^+^ or TUNEL^+^ RGCL NeuN^+^ neurons in two central retinal sections per globe were counted by a blinded observer. Cell counts were normalized to length of RGCL as determined using ImageJ software.

### Statistical Analysis

Where applicable, multiple comparisons were performed by one or two way analysis of variance (ANOVA) followed by Bonferroni *post* test or student's unpaired *t* test using GraphPad Prizm software (San Diego, CA). Statistical significance was assumed if *P*<0.05. Values were graphically represented as mean ± SEM from all individuals in each group of animals.

## Results

### BMT Results in Robust Donor Microglia Engraftment and Normalization of Total Microglia in APP*swe*-PS1ΔE9 Retina

**Figure 2:**
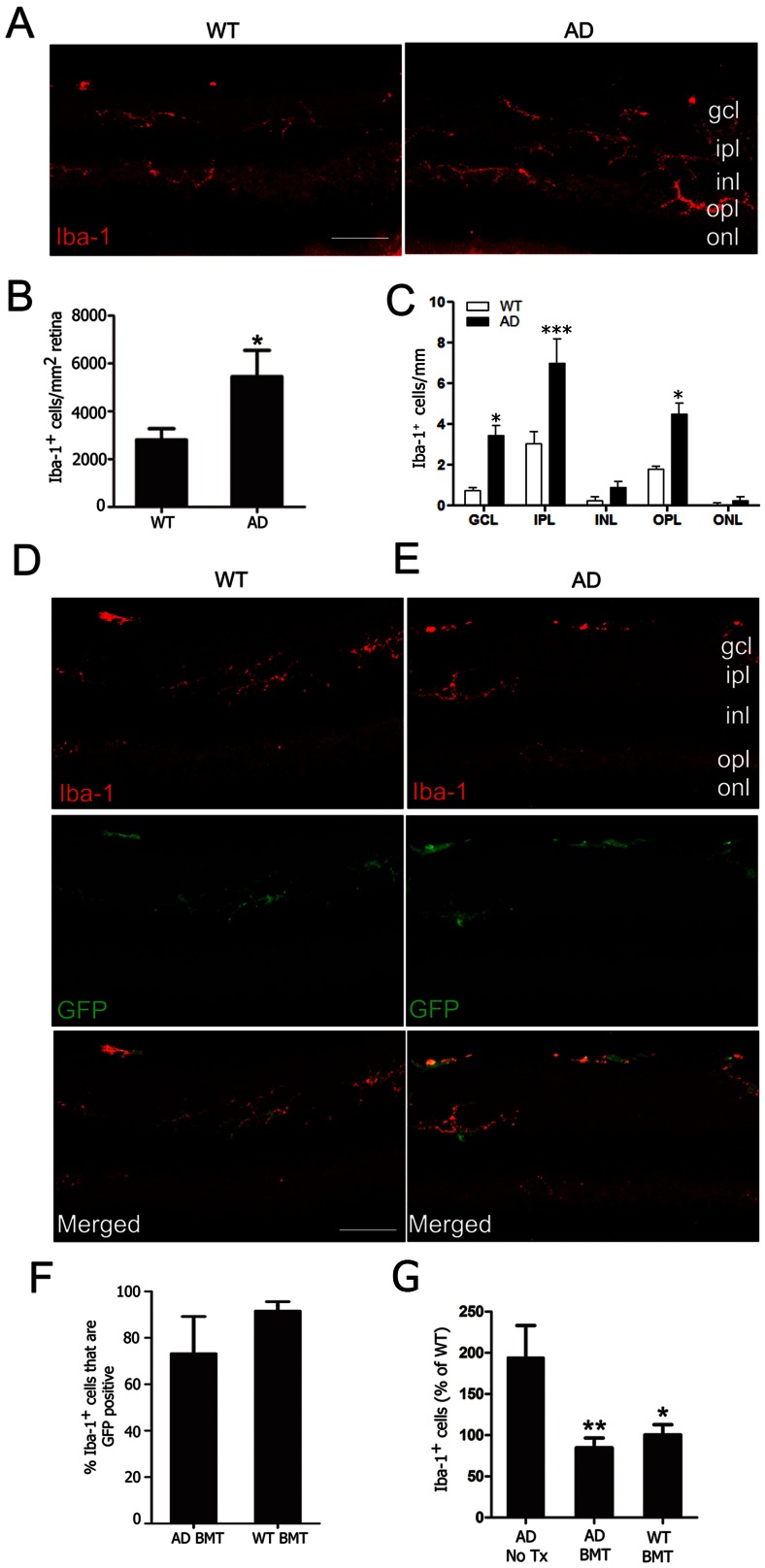
Increased microglia density in experimental AD is mitigated by BMT. **A**: Representative retinal cryosections from wt (left) and APP*swe*-PS1ΔE9 mice (right) were stained with anti-Iba-1 antibody and visualized with Cy3-conjugated secondary antibody. Ramified microglia were primarily identified in ganglion cell layer (gcl), inner plexiform layer (ipl) and outer plexiform layer (opl) in wt and APP*swe*-PS1ΔE9 retinas. **B**: Unbiased stereologic analysis revealed increased microglia density in control (untreated) APP*swe*-PS1ΔE9 retina compared with wt control mice (*P<0.05, n = 7–9, student's *t* test). **C**: Average Iba-1^+^ microglia density in the different retinal layers. Significantly higher numbers of Iba-1^+^ microglia were noted in gcl, ipl and opl in APP*swe*-PS1ΔE9 mice (*P<0.05, ***P<0.001, n = 7–9, two-way ANOVA followed by Bonferroni *post* test.) Confocal images from wt (**D**) and APP*swe*-PS1ΔE9 (**E**) retinas immunostained with Iba-1 (red) demonstrate that BM-derived GFP^+^ cells (green) exhibited ramified microglia morphology and had near-uniform expression of Iba-1. In both wt and APP*swe*-PS1ΔE9 BMT recipient mice, host microglia were almost completely replaced with BM-derived cells. **F**: Unbiased stereologic quantitation of retinal microglia engraftment revealed 91.2±4.0% of wt and 73.2±16.0% of APP*swe*-PS1ΔE9 Iba-1^+^ microglia were BM derived (GFP^+^). **G**: Microglia density in retina of wt BMT recipients was not different from non-transplanted wt mice, but BMT in APP*swe*-PS1ΔE9 mice normalized microglia density to wt levels. Data are percent of wt Iba-1^+^ cells/mm^2^ in non-transplanted APP*swe*-PS1ΔE9 untreated mice (AD no Tx) or APP*swe*-PS1ΔE9 and wt BMT recipients (*P<0.05, **P<0.01, n = 6–10, one-way ANOVA followed by Bonferroni *post* test). Scale bars  = 30 μm.

Microglia are the principle innate immune effector cells in brain and retina and are implicated in Aβ-related retinal degeneration [25], [26]. We hypothesized that BMT-mediated mitigation of pathologic changes in AD retina would necessarily require engraftment of transplanted cells. However, in order to understand the effects of BMT on resident microglia, we first characterized Iba-1 immunopositive microglia in non-transplanted, aged (13-month-old) wt and APP*swe*-PS1ΔE9 control retina. Iba-1 is a calcium binding adaptor protein that is expressed in monocytes, macrophages and microglia. Iba-1 immunopositive cells in wt and APP*swe*-PS1ΔE9 retina exhibited a range of morphologies from classically ramified (small soma and multiple long delicate processes) to reactive (cytoplasmic enlargement and fewer, coarser processes) (Fig. 2A). In agreement with previously published observations, microglia in APP*swe*-PS1ΔE9 mice were identified in outer plexiform layer in addition to layers of inner retina normally populated with microglia [22]. Stereologic quantification of Iba-1^+^ cells revealed 48.5±19.9% more Iba-1^+^ cells in APP*swe*-PS1ΔE9 retina compared with wt (Fig. 2B, *P*<0.05, student's *t* test). Quantification of Iba-1^+^ microglia in different retinal layers demonstrated significantly more Iba-1^+^ cells in RGCL, inner plexiform layer and outer plexiform layer of APP*swe*-PS1ΔE9 compared with wt mice (Fig. 2C, *P<0.05, ***P<0.001, two-way ANOVA with Bonferroni *post* test).

We next evaluated BMT-recipient mice for engraftment of donor cells by quantifying the amount of total Iba-1^+^ microglia that were also GFP^+^. Photomicrographs from representative wt (Fig. 2D) and APP*swe*-PS1ΔE9 (Fig. 2E) mice that received wt;GFP BMT demonstrate similar Iba-1^+^ microglia morphology, density and distribution between host strains. BM-derived cells were located predominantly in the ganglion cell and inner plexiform layers in both strains, consistent with the observations of others [43]. BM-derived microglia engraftment was remarkable in both wt (91.2±4.0%) and APP*swe*-PS1ΔE9 (73.2±16.0%) recipients. There was no significant difference between recipient strains although variability was increased in APP*swe*-PS1ΔE9 BMT recipients (Fig. 2F). These findings are broadly consistent with those of other investigators who described robust long-term BM-derived cell engraftment in mouse retina after whole body irradiation [43]. We quantified total retinal microglia in BMT-recipients in order to determine whether BMT cells replaced or supplemented resident microglia and found, surprisingly, that total microglia in wt and APP*swe*-PS1ΔE9 BMT-recipient mice were significantly lower than non-transplanted control APP*swe*-PS1ΔE9 retina but not significantly different from non-transplanted wt retina (Fig. 2G), indicating that BMT resulted in normalization of total microglia to non-disease levels.

### BMT Reduces Aβ and PHF-tau Immunofluorescence in APP*swe*-PS1ΔE9 Retina

**Figure 3:**
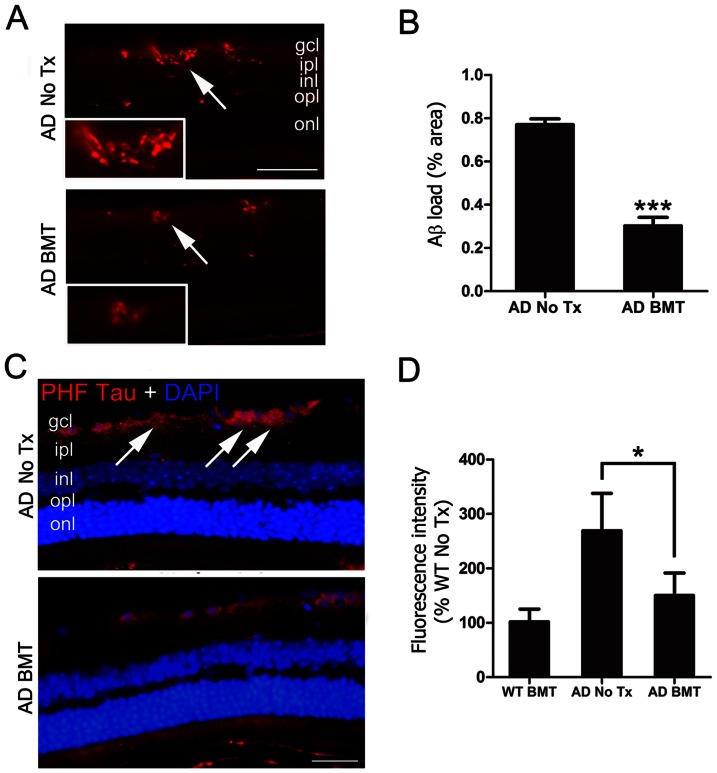
Aβ and PHF-tau are reduced in retina of APP*swe*-PS1ΔE9 BMT recipient mice. **A**: Representative photomicrographs of Aβ deposition in non-transplanted, age-matched APP*swe*-PS1ΔE9 control retina (top, AD No Tx) or APP*swe*-PS1ΔE9 that received BMT (bottom, AD BMT) stained with anti-Aβ antibody and visualized with Cy3-conjugated secondary antibody (red). Region of inset is indicated by arrows. Scale bar  = 50 µm. **B**: Quantitative analysis of Aβ immunofluorescence using a standardized digital thresholding protocol demonstrated significant reduction in retinal Aβ in BMT APP*swe*-PS1ΔE9 mice compared with non-transplanted APP*swe*-PS1ΔE9 control mice (***P<0.001, n = 6, student's *t* test). **C**: Representative photomicrographs of PHF-tau immunofluorescence in retinal ganglion cell layer (RGCL) (arrows) of non-transplanted, age-matched control APP*swe*-PS1ΔE9 mice (top, AD No Tx) compared with APP*swe*-PS1ΔE9 BMT recipients (bottom, AD BMT). Nuclei were counterstained with DAPI (blue). Scale bar  = 30 µm. **D**: Quantitative analysis of PHF-tau immunofluorescence using a standardized digital thresholding protocol demonstrated significant reduction of PHF-tau in APP*swe*-PS1ΔE9 BMT-recipients compared with non-transplanted controls (*P<0.05, n = 6, one-way ANOVA analysis with Bonferroni *post* test).

The pathologic hallmarks of AD brain are Aβ deposits in the form of neuritic plaques and tau aggregation in the form of neurofibrillary tangles (NFT), but neither of these classical structures has been described in human or mouse retina. In agreement with others, we also did not identify neuritic plaques or NFT in APP*swe*-PS1ΔE9 retina. However, in agreement with previous reports, immunofluorescence stains of non-transplanted 13-month-old APP*swe*-PS1ΔE9 mice revealed extensive Aβ deposition with numerous focal plaque-like densities in a background of variably intense immunoreactivity that was mainly present in the RGCL and inner > outer plexiform layers (Fig. 3A, upper panel). A representative photomicrograph of an APP*swe*-PS1ΔE9 BMT-recipient retina (Fig. 3A, lower panel) shows a clear and significant reduction in Aβ that on average was 60.7±5.8% less than non-transplanted controls (Fig. 3B) and was even more effective than BMT-mediated reduction of Aβ in brain. The pathological counterpart to Aβ in AD is the accumulation of intracellular PHF-tau, which is neurotoxic [44], [45]. Previous studies have demonstrated hyperphosphorylated tau in the APP*swe*-PS1ΔE9 mouse brain [46], so we evaluated PHF-tau immunoreactivity in APP*swe*-PS1ΔE9 retina. We found intense intracellular staining predominantly localized to RGC soma and processes in non-transplanted 13-month-old APP*swe*-PS1ΔE9 mice (Fig. 3C, upper panel). BMT resulted in reduced RGCL PHF-tau as demonstrated by a representative photomicrograph (Fig. 3C, lower panel). Quantitative immunofluorescence analysis confirmed significantly reduced PHF-tau in APP*swe*-PS1ΔE9 BMT-recipients compared with age-matched APP*swe*-PS1ΔE9 controls (*P*<0.05, one-way ANOVA with Bonferroni *post* test) and no difference between BMT-recipient APP*swe*-PS1ΔE9 mice and non-transplanted or BMT-recipient wt mice (Fig. 3D).

### Retinal Ganglion Cell Neuroprotection in Aged WT and APP*swe*-PS1ΔE9 BMT Recipients

**Figure 4:**
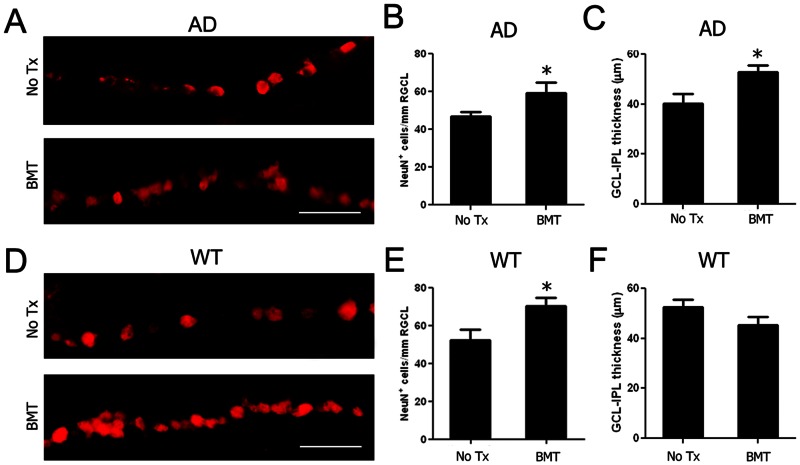
BMT mediates neuroprotection of RGCL neurons in APP*swe*-PS1ΔE9 and wt mice. **A–C**: RGCL neurons were identified using anti-NeuN antibody and visualized with Cy3-conjugated secondary antibody in 13-month-old APP*swe*-PS1ΔE9 and wt mice. Representative photomicrographs of NeuN^+^ RGCL neurons in control (A, top) and BMT-recipient (A, bottom) APP*swe*-PS1ΔE9 mice demonstrate neuroprotective effects of BMT through preservation of RGCL neurons (B) and inner retinal (NFL+RGCL+IPL) thickness (C). **D–E**: Representative photomicrographs of NeuN^+^ RGCL neurons in 13-month-old control (D, top) and BMT-recipient (D, bottom) wt mice also demonstrate neuroprotective effects of BMT through preservation of RGCL neurons (E). **F**: No effects on retinal thickness in wt recipients compared with the wt controls. *P<0.05, n = 6–10, student's *t* test. Scale bar  = 50 μm.

Retinal neuronal degeneration is associated with Aβ deposition in AD [47], [48] and loss of retinal neurons in GCL has been demonstrated in both clinical and experimental AD [9], [49]. We hypothesized that BMT-mediated Aβ reduction would result in neuroprotection in retina, so we evaluated RGCL neuron survival in non-transplanted and BMT recipient APP*swe*-PS1ΔE9 mice using immunofluorescence. Representative photomicrographs of RGCL of age-matched control (Fig. 4A, upper panel) and 13-month-old BMT-recipient (Fig. 4A, lower panel) APP*swe*-PS1ΔE9 mice stained with anti-NeuN antibody demonstrate increased NeuN^+^ neurons in BMT-recipients. Quantification of total RGCL neurons showed significant preservation with BMT (Fig. 4B) (*P*<0.05, student's *t* test). We also quantified RGCL neurons in age-matched control (Fig. 4D, upper panel) and 13-month-old BMT-recipient (Fig. 4D, lower panel) wt mice and, to our surprise, found similar neuroprotective effects in RGCL density (Fig. 4E) (*P*<0.05, student's *t* test) as in APP*swe*-PS1ΔE9 mice. Indeed, RGCL neuron density was not significantly different between untreated APP*swe*-PS1ΔE9 and wt mice at 13 months of age.

This was not true, however, for inner retina diameter. Reduced retinal thickness, a manifestation of neuron loss, has been detected in patients with AD [50], [51], [52]. As a correlate to human disease, we found that the inner retinal (NFL + RGC + IPL) thickness was significantly reduced in aged-matched, untreated APP*swe*-PS1ΔE9 mice compared with 13-month-old wt mice (Fig. 4C and 4F, *P*<0.05) which suggests that, unlike RGCL neurons, inner retinal diameter did appear to be associated with expression of the APP*swe*-PS1ΔE9 transgene. Inner retinal thickness from APP*swe*-PS1ΔE9 mice that received BMT was significantly increased compared with APP*swe*-PS1ΔE9 controls (Fig. 4C, *P*<0.05) and was not significantly different from age-matched, untreated wt mice (Fig. 4F, *P*>0.05). Interestingly, at 13 months of age, BMT recipient mice inner retinal diameter trended toward a reduction compared with untreated, age-matched controls, but this difference did not achieve statistical significance (Fig. 4F). Overall, these results demonstrate neuroprotective effects of BMT in inner retinal neurodegeneration and suggest that non-Aβ-dependent processes preferentially impact RGCL neurons while Aβ neurotoxicity is mediated at the level of the neuropil.

We hypothesized that BMT-mediated neuroprotection of RGCL neurons was a result of decreased neuron death. To assess this, apoptotic neurons in RGCL were evaluated with double NeuN/TUNEL immunostaining, but TUNEL^+^ cells were very rare and no significant differences were identified between control and BMT recipient groups (data not shown). As an alternative explanation, we hypothesized that RGC neurogenesis, although normally dormant in adult retina, could be induced by BMT. However, NeuN^+^ RGCL neurons co-labeled with antibodies to BrdU were extremely rare in both groups and there was no statistically significant difference between control and BMT recipients (data not shown). It is not surprising that TUNEL rates are low in a chronic condition that requires months if not years to progress. By the same token, it is well-known that neurogenesis of adult RGCs is at best a very rare event. Thus, it is likely that our study was not powered sufficiently to detect differences in either process and thus neither can be confidently excluded. However, based on published rates of adult RGC neurogenesis, we favor BMT-mediated prevention of cell loss, through prevention of apoptosis or other cell death pathways.

**Figure 5:**
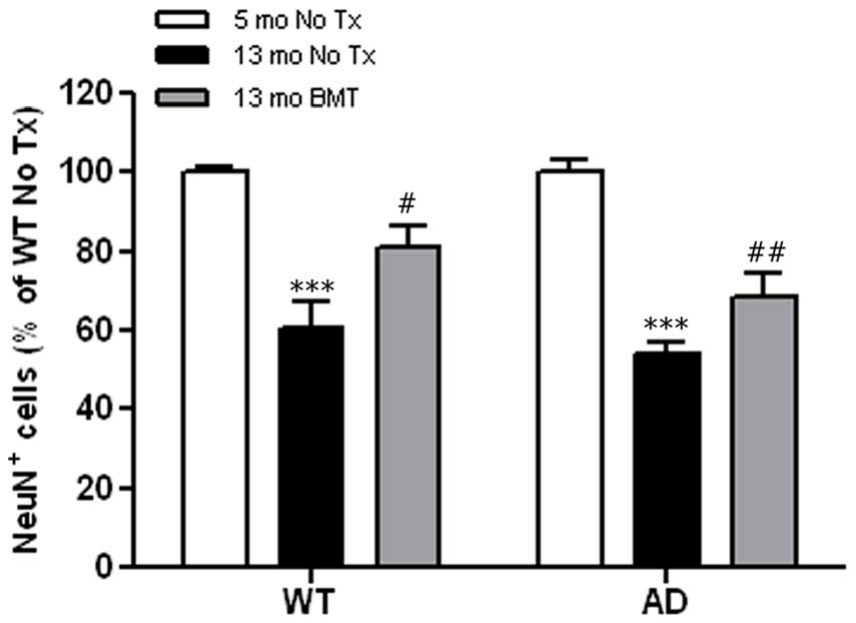
Age-related RGCL neurodegeneration is mitigated by BMT in wt and APP*swe*-PS1ΔE9 mice. NeuN^+^ RGCL neuron density was compared between 13-month-old BMT recipient mice and non-transplanted young (5 mo) and age-matched (13 mo) wt and APP*swe*-PS1ΔE9 mice and presented as percent of 5-month-old wt non-transplanted controls. There was a significant age-dependent reduction in neuron density that was partially rescued in wt and APP*swe*-PS1ΔE9 BMT recipient mice. ***P<0.001, ^#^ *P*<0.05, ^##^ *P*<0.01, n = 6–10, two-way ANOVA followed by Bonferroni *post* test.

It was clear that BMT-mediated neuroprotection was present in both wt and APP*swe*-PS1ΔE9 mice. We hypothesized that this neuroprotection may be a result of decreased age-related neurotoxicity based on a recent study of human retina that identified significant loss of RGCs in aged vs. young retinas [2]. To test this possibility, we examined retinas from young (5-month-old) wt and APP*swe*-PS1ΔE9 mice and found approximately 40% more RGCL neurons in eyes from young mice of both genotypes compared with 13-month-old wt and APP*swe*-PS1ΔE9 controls in the absence of transplantation, irradiation, or other manipulations (Fig. 5, *P*<0.001, two-way ANOVA Bonferroni *post* test). Our data suggest that the neuroprotective effects of BMT target age-related RGCL neuron loss independent of Aβ, and that BMT provides added protection against Aβ-mediated loss of inner retinal neuropil.

### Irradiation has No Protective Effect on RGCL Neurons

**Figure 6:**
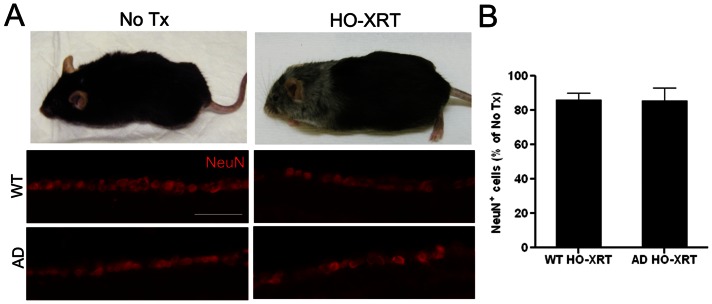
RGCL neuroprotection is not due to effects of high dose cranial irradiation alone. **A**: Representative photographs of control mice (no Tx) and mice that received head only irradiation (HO-XRT) demonstrate effects of irradiation on coat color and confirm radiation exposure in HO-XRT mice (top). Representative photomicrographs of NeuN^+^ neurons (red) in RGCL stained with NeuN antibody and visualized with Cy3-conjugated secondary antibody show a mild reduction in neuron density 8 months after HO-XRT (bottom). Scale bar  = 50 μm. **B**: Quantification of neuron density depicted as a percent of age-matched, non-irradiated wt controls demonstrates there is a mild reduction in RGCL neurons as a result of high dose irradiation without BMT in wt and APP*swe*-PS1ΔE9 mice, thus eliminating the possibility that high dose cranial irradiation underlies the neuroprotective effects of BMT.

Previous studies in our lab demonstrated that high dose cranial irradiation is not sufficient to reduce the accumulation of Aβ peptides or plaques in APP*swe*-PS1ΔE9 mice cerebrum [32]. In order to exclude an effect of high dose cranial irradiation in the observed protection of retina, we exposed 5-month-old wt and APP*swe*-PS1ΔE9 mice to the same dose of irradiation given to BMT recipients (10.5 Gy) but shielded the body from the neck to the tip of the tail. These “head only” irradiated mice did not receive BMT and were euthanized 8 months post-irradiation at 13 months of age. Grayish discoloration of the fur in irradiated mice confirmed complete cranial irradiation to the exclusion of the rest of the body (Fig. 6A). Quantification of NeuN^+^ cells was performed in a manner identical to previous experiments (Fig. 4) and revealed mildly reduced NeuN^+^ RGCL neurons in wt and APP*swe*-PS1ΔE9 mice that received cranial irradiation compared with non-irradiated controls (Fig. 6A and 6B). While others have recently demonstrated neuroprotective effects of irradiation in experimental glaucoma [53], [54] and retinitis pigmentosa [55], and evidence of irradiation-induced RGCL neuron toxicity is relatively limited to developing retina [56], [57], [58], [59], we believe this is the first study to show no protective effect of irradiation in normal aging and experimental AD.

### Innate Immune Response Is Enhanced with BMT

**Figure 7:**
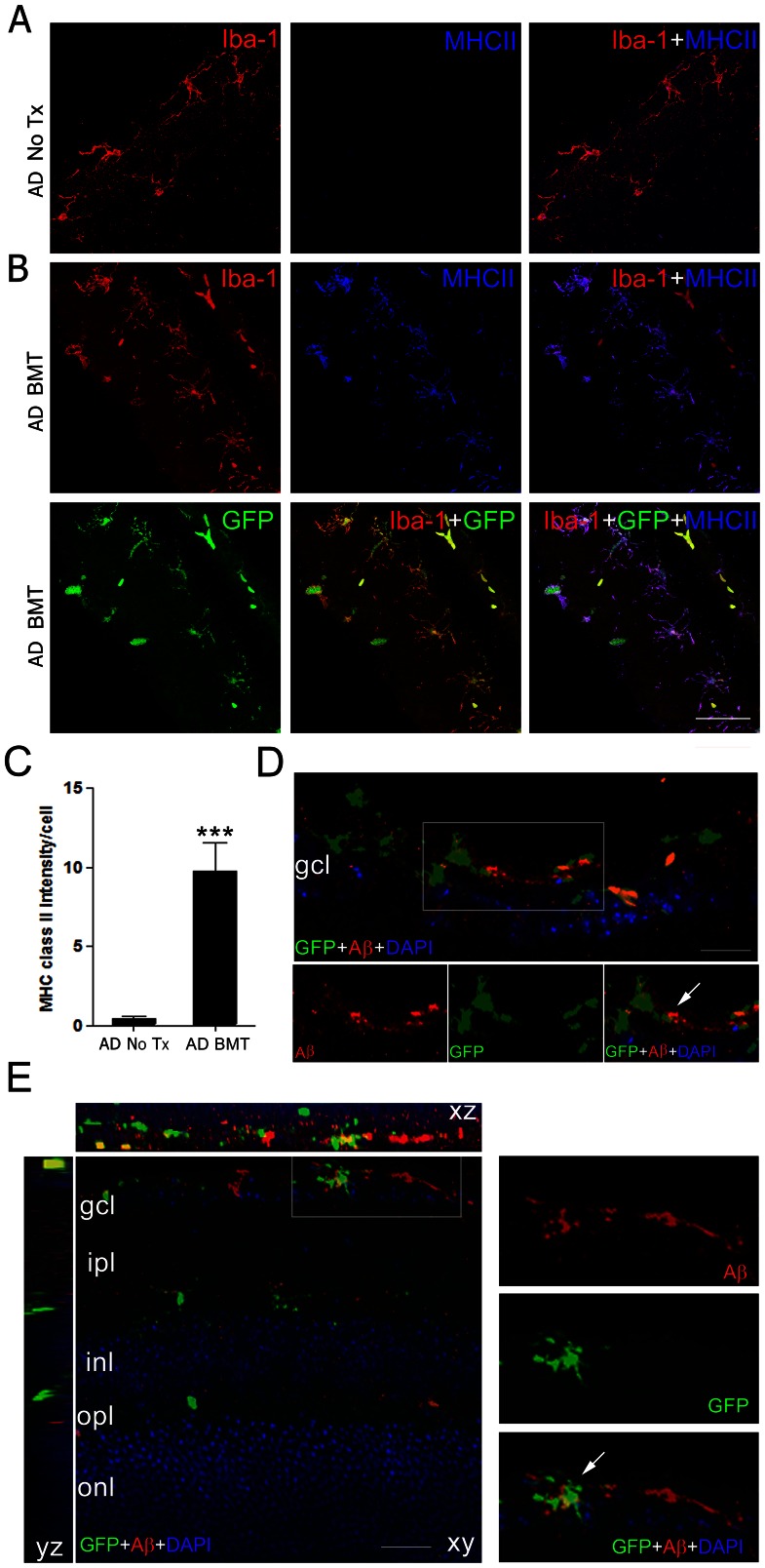
MHC class II is up-regulated in BMT-derived retinal microglia. **A**: Representative photomicrographs of microglia in the retina of a 13-month-old non-transplanted wt mouse stained with anti-Iba-1 antibody and visualized with Cy3-conjugated secondary antibody (red). The endogenous Iba-1^+^ microglial cells do not express detectable MHC class II by immunofluorescence stains (blue, overlay with Cy3 fluorescence) in untreated (No Tx) wt retina. **B**: Representative photomicrographs of retinal sections from a 13-month-old wt mouse transplanted with GFP^+^ BM cells. Immunofluorescence staining demonstrates that the Iba-1^+^ microglia (red) are almost completely derived from the BMT (green GFP^+^ cells). GFP^+^ cells were strongly immunoreactive for MHC class II (blue). The overlay of the confocal microscope images indicates co-expression of GFP (green), Iba-1 (red) and MHC class II (blue) in retina. Scale bar  = 30 μm. **C**: Quantification of MHC class II immunofluorescence in microglia shows significantly increased expression in BM-derived cells compared with the endogenous microglia. ***P<0.001, n = 6, student's *t* test. **D**: Confocal analysis of sections demonstrates association of BM-derived microglia (GFP^+^, green, arrow) and Aβ deposits (red). High magnification of inset is shown on the lower panels. Scale bar: 20 µm. **E**: 3D-Confocal image analysis of intracellular Aβ in BM-derived microglia. The overlay of confocal images reveals Aβ deposition (red) within GFP^+^ BM-derived microglia (green, arrow). High magnification of inset is shown on the right panels. Scale bar: 20 µm.

In agreement with previous studies [43], we found robust engraftment of BM-derived microglia in retina (Fig. 2); non-microglia lineage cells, including neurons and other glia, were uniformly of host origin. Based on this, we hypothesized that BMT-mediated neuroprotection was most likely secondary to modulation of innate immune related proteins. MHC class II expression in microglia is associated with increased Aβ clearance in APP*swe*-PS1ΔE9 mice [34] and is increased in BMT-derived microglia in brain (personal observation). Thus, since we found significantly reduced retinal Aβ in BMT-recipient APP*swe*-PS1ΔE9 mice (Fig. 3), we hypothesized that BMT-derived microglia would exhibit increased MHC class II. Immunofluorescence staining revealed weak expression in Iba-1^+^ cells in non-transplanted controls (Fig. 7A). However, in BMT-recipient mice, strong MHC class II expression was found in the GFP^+^ BM-derived cells compared with rare endogenous (GFP^−^) microglia (Fig. 7B). Fluorescence intensity analysis revealed significantly increased MHC class II expression in BM-derived cells compared with endogenous microglia (*P*<0.001, Student's *t*-test, Fig. 7C). MHC class II expression is dependent on phagocytosis of extracellular proteins, such as Aβ, and may be increased in a pro-inflammatory innate immune response, although evidence for this is inconsistent [60]. Confocal image analysis of adjacent sections confirmed BM-derived microglia cells contained intracellular Aβ immunoreactivity and had processes extending into immunopositive amyloid (Fig. 7D and 7E). Thus, MHC class II is up-regulated on BMT-derived microglia that contain intracellular Aβ.

**Figure 8:**
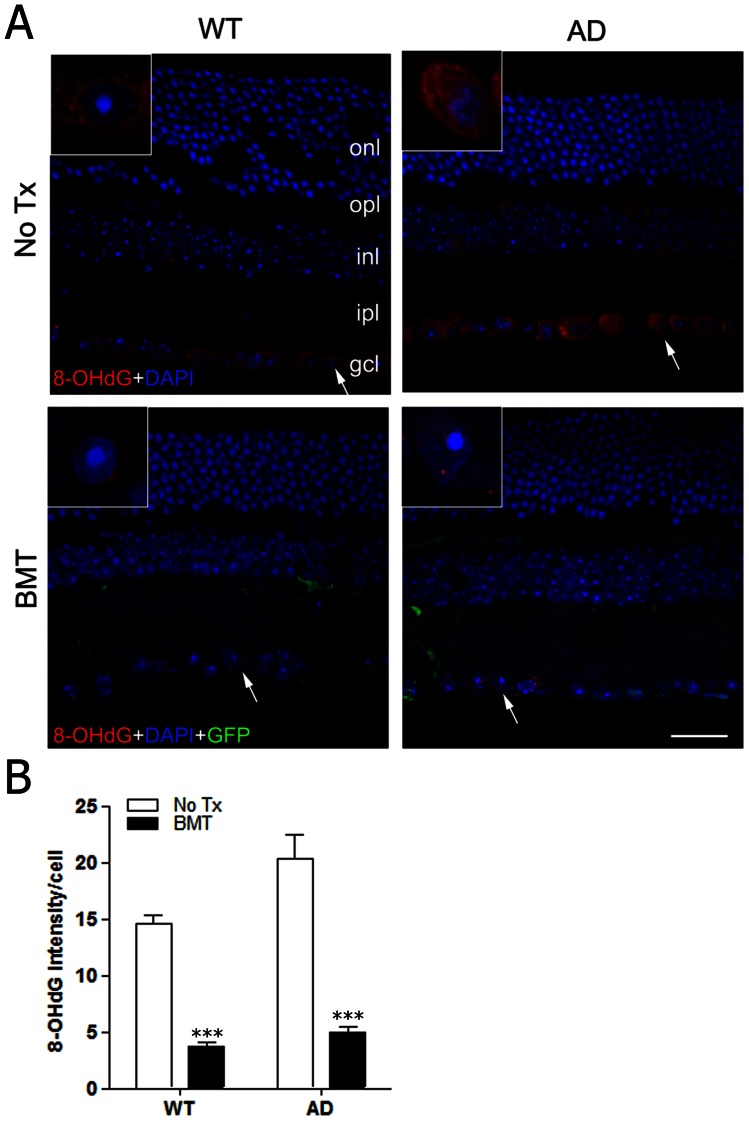
BMT results in reduced RGCL oxidative stress in aged wt and APP*swe*-PS1ΔE9 mice. **A:** Immunofluorescence stains for 8-OHdG (red), an indicator of oxidative stress, are shown in representative retinal cross-sections from age-matched wt (left column) or APP*swe*-PS1ΔE9 (right column) mice that received no BMT transplant (top row) or BMT (bottom row). 8-OHdG immunofluorescence is primarily detected in RGCL neurons in non-transplanted wt and APP*swe*-PS1ΔE9 mice in a diffuse, perikaryal pattern. However, only focal, punctate immunostaining was observed in retinas from wt and APP*swe*-PS1ΔE9 mice that received BMT. Arrows indicate regions highlighted in insets. Scale bar  = 20 μm. **B:** Quantification of 8-OHdG immunofluorescence relative intensity in RGCL neurons confirmed a significant reduction in 8-OHdG immunostaining in wt and APP*swe*-PS1ΔE9 mice that received BMT compared with non-transplanted controls, respectively. ***P<0.001, n = 6–10, two-way ANOVA followed by Bonferroni *post* test.

Whether pro- or anti-inflammatory, microglia activation is a complex process which depends on selective elaboration of a diverse repertoire of cytokines, chemokines, proteases, and prostanoids that can be neuroprotective or neurotoxic depending on the subset of molecules secreted. Because our primary endpoint, immunohistology, required fixed tissues, we were unable to quantitatively assay for the aforementioned molecules, and immunostains were uninformative (data not shown). However, a ubiquitous endpoint of classical, pro-inflammatory innate immune activation is oxidative damage mediated by elaboration of microglial ROS. Alternatively, microglia contain multiple antioxidative defense mechanisms including abundant glutathione, superoxide dismutase, catalase, and other enzymes that can mitigate oxidative stress [61]. It is well-accepted that age-related neurodegeneration may be due to increased DNA damage caused by oxidative stress with age [62]. In order to determine the effects of BMT on oxidative stress in retinal neurons, we analyzed retinal sections for the presence of 8-hydroxydeoxyguanosine (8-OHdG), an indicator of oxidative DNA damage, in aged (13-month-old) wt and APP*swe*-PS1ΔE9 non-transplanted and BMT recipient mice. 8-OHdG is prominent in RGCL neurons in control wt and APP*swe*-PS1ΔE9 retina, but was markedly decreased in RGCL neurons of BMT recipient mice (Fig. 8A). Quantitative analysis revealed significantly reduced 8-OHdG relative intensity in RGCL of wt and APP*swe*-PS1ΔE9 mice received BMT compared with non-transplanted age-matched controls, respectively (Fig. 8B, *P*<0.001, two way ANONA using Bonferroni *post hoc* test). We interpret this data to indicate that BMT shifted aging retina from a neurotoxic, pro-inflammatory, oxidative environment to a neuroprotective, alternatively activated, pro-phagocytic, and anti-oxidative milieu that resulted in reduced Aβ and preservation of RGCL neurons.

## Discussion

We have further characterized the pathologic changes of experimental aging and AD in retina and found a strong effect of age on retinal neurodegeneration that was mitigated by BMT. Although BMT led to reduced retinal Aβ and PHF-tau and normalized total microglia, the pathologic effects of AD on RGCL neuron survival were small compared with the effects of normal aging. We provide evidence for BMT-mediated neuroprotection of inner retina in aging and experimental AD that is mediated through altered microglia innate immune response, resulting in decreased oxidative damage to RGCL neurons, and demonstrate that high dose cranial irradiation is not sufficient to mediate this RGCL neuroprotection in the absence of BMT.

In this study we chose to evaluate cross-sections of retina rather than whole mount specimens. While whole mount retinas provide complete cytoarchitectural details and are very useful for morphologic analysis of cells, this study was designed with histologic sections in order to perform Iba-1, Aβ, PHF-tau, MHC class II, BrdU, and 8-OHdG immunostains as well as TUNEL assays. While histologic sections limit analyses to part of the whole retina, systematic sampling was performed at the level of the optic nerve in this study to limit bias in this region. Future studies with BMT-recipient APP*swe*-PS1ΔE9 mice will be valuable to assess whole mount preparations to address cytoarchitectural features. This study is also limited by the lack of a true irradiation control, since myeloablative whole body irradiation without a BMT is lethal. We attempted to address this critical point by performing head only irradiation in APP*swe*-PS1ΔE9 mice, which resulted in a mild (∼10%) reduction in RGCL neurons in wt and APP*swe*-PS1ΔE9 retina compared with non-irradiated controls. This control experiment suggests that direct high dose radiation alone is not neuroprotective, but the effects of high dose, whole body irradiation in the absence of BMT cannot be elucidated using presently available technologies and model systems.

Previous reports have characterized engraftment of BM-derived cells from GFP-expressing donor mice in the retinas of wt recipients with mixed results. Xu *et al* [37] showed that nearly all retinal microglia were BM-derived by 6 months post-BMT, and Boettcher *et al* [43] found incomplete replacement of retinal microglia by BM-derived cells up to 15 months post-transplantation. In this study, we found approximately 91% engraftment of BM-derived microglia in wt recipients with more variability in APP*swe*-PS1ΔE9 recipients (∼73% engraftment), confirming robust replacement of retinal microglia after BMT. There was no significant difference in total BM-derived microglia between wt and AD host mice, but the trend suggests microglia replacement in diseased (APP*swe*-PS1ΔE9) retina, unlike our previous studies in brain [32], may be similar or even less efficient when compared with healthy controls. Interestingly, total APP*swe*-PS1ΔE9 retinal microglia normalized to wt levels with BMT.

It is interesting biologically and from a therapeutic perspective that BMT resulted in near complete replacement of endogenous microglia with phenotypically distinct BM-derived cells. In the earliest experimental BMT studies that focused on CNS engraftment in irradiated mice, both perivascular macrophages and microglial cells were found to be replaced by BM-derived cells [35], [63], [64]. These BM-derived cells expressed Iba-1 but were functionally different from endogenous microglia by their distinctive phenotype [31], [65], [66]. We confirmed these studies in brain by demonstrating distinctive up-regulation of MHC class II in BM-derived cells compared with endogenous microglia, and have identified these cells in both perivascular and parenchymal distributions. APP*swe*-PS1ΔE9 retinal microglia are more abundant. Our data suggest that the number of activated microglia increases in response to Aβ and this is normalized by BMT.

BMT-derived microglia engraftment appears to be more efficient in retina than in brain in wt mice but not in experimental AD. WT BMT into wt recipients resulted in >90% engraftment of donor-derived microglia 8 months post-transplant, which is remarkable considering only ∼50% engraftment in the brain over the same period of time in the same mice [67]. This supports previous observations that BM-derived monocyte precursor cells are able to efficiently migrate across the blood-retina barrier (BRB) and replace endogenous microglia [37]. It was recently shown that a 10 Gy dose of irradiation did not result in significant histological changes in the mouse retina [56], but microscopic or ultrastructural changes to retina may make the BRB more sensitive to radiation preconditioning than the blood brain barrier (BBB). Alternatively, the BRB or retinal parenchyma may be inherently more favorable for blood monocyte migration and engraftment than the brain, or BM-derived monocytes have enhanced capacity for migration in retina.

Previous studies have shown high dose irradiation accompanied with BMT can confer complete protection against glaucoma in a mouse model [68]. To our knowledge, the studies described here are the first to demonstrate BMT-mediated alterations in Aβ peptides in APP*swe*-PS1ΔE9 retina. Previous studies have described the formation of Aβ plaques in the retina of APP*swe*-PS1ΔE9 mice, which exhibit a similar chemical phenotype to those observed in brain [22]. BMT-mediated Aβ-reduction could occur via inhibition of Aβ production or enhanced clearance. We favor the latter since previous studies have demonstrated enhanced phagocytosis by BM-derived cells compared with resident microglia [29], [69]. We cannot exclude an independent effect of whole body irradiation on Aβ levels because the appropriate controls (myeloablative whole body irradiation without BMT) cannot be performed due to lethality. While, high dose cranial irradiation without BMT has been previously shown to have no effect on cerebral Aβ in APP*swe*-PS1ΔE9 mice [32], and we show no effect of cranial irradiation without BMT on RGCL neuron survival, there is no clear methodology by which the specific role of high dose whole body irradiation can be discerned. Seminal studies [70] showed cerebral engraftment of BM-derived cells required a preconditioning therapy, such as irradiation. While this does not address the specific role of whole body, myeloablative irradiation in retinal neuroprotection, it supports the idea that BMT-mediated neuroprotection involves effects of preconditioning therapy as well as the transplanted cells themselves.

Although neuroprotection can be induced by physical and pharmacologic disruption of eye structures [71] and by varying doses of irradiation in other models of eye diseases such as glaucoma [53], [54] and retinitis pigmentosa [55], we found no protective effect on RGCL neuron loss in aging or experimental AD in mice that received high dose cranial irradiation. The reason for this discrepancy may be related to comparisons made between disparate pathologic processes (aging and AD vs. glaucoma and retinitis pigmentosa), methodologies, animal strains/species, and/or outcome measures. Future experiments to further characterize the neuroprotective effects of irradiation and BMT are necessary to better understand the mechanisms of both applications.

Accumulation of Aβ deposits may influence hyperphosphorylation of tau protein [23], [72], [73], PHF-tau mediated pathogenic mechanisms appear to be involved in neurodegeneration of retina, as suggested by elevated phosphorylated tau in the optic nerve of patients with retinal degenerative disease [74]. In this study, we found increased PHF-tau associated with Aβ deposition within the retina of APP*swe*-PS1ΔE9 mice that was reduced by BMT. However, the molecular mechanisms by which BM-derived microglia promotes Aβ clearance and PHF-tau attenuation are not fully understood and require further investigation in both brain and retina.

Previous studies of AD found reduced thickness of the nerve fiber layer [75]; we identified a significant increase in inner retinal thickness in APP*swe*-PS1ΔE9 retina following BMT. In AD, Aβ deposits directly disrupt the organization of the retinal neuropil [22]. We hypothesize that reduced Aβ in BMT recipients resulted in preserved inner retinal parenchyma, a sensitive measure of retinal neurotoxicity. However, the effects of Aβ peptides on RGCL neuron populations are unresolved. While some studies have shown no significant loss of RGC in AD patients [9], others report substantial RGC loss [76]. We found ∼10% reduction in RGCL neurons in APP*swe*-PS1ΔE9 mice compared with age matched wt controls that was not statistically significant. Presumably, APP*swe*-PS1ΔE9 RGCL neuron pathology progresses with age and therefore older mice might exhibit more pronounced pathology. APP*swe*-PS1ΔE9 and wt BMT recipients had significantly more RGCL neurons compared with non-transplanted controls, and there was a significant age-related degeneration of RGCL neurons in APP*swe*-PS1ΔE9 and wt mice. We interpret this finding to indicate that Aβ-neurotoxicity exacerbates age-related degenerative pathways. Oxidative stress is one of the principle mechanisms of neuronal death during normal aging and age-associated disorders. Here we found the 8-OHdG levels were significantly decreased in neurons of the retina from BMT recipient mice compared with the controls, suggesting BM-derived cells mitigate oxidative damage to neurons in age associated retinal degeneration.

**Figure 9:**
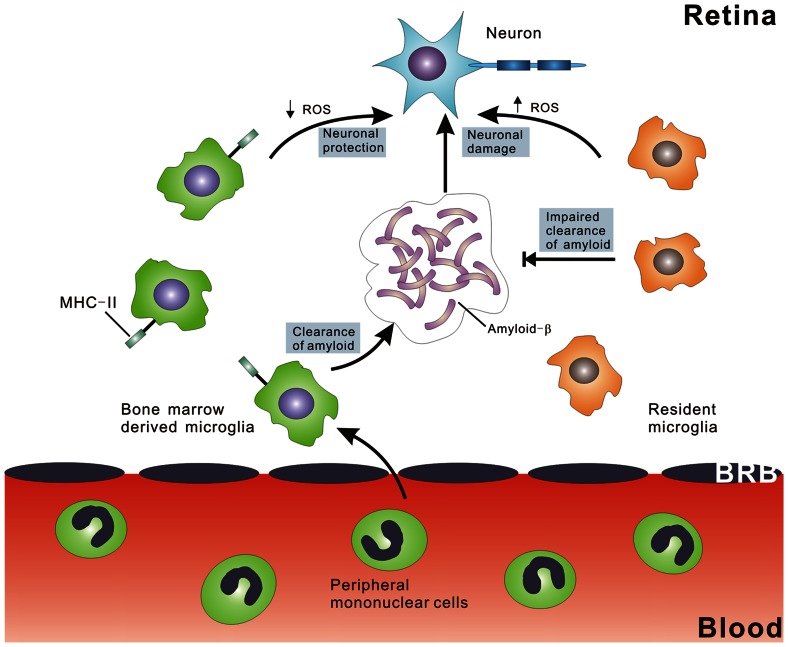
Schematic representation of the mechanism of BMT-mediated neuroprotection in neurodegeneration of inner retina. Endogenous microglia (right side) have a pro-inflammatory phenotype that results in impaired phagocytosis of Aβ and increased elaboration of ROS, resulting in increased neurotoxicity. In contrast, BM-derived peripheral blood monocytes migrate into the retina through the blood retinal barrier, and then differentiate into microglia characterized by MHC class II expression. This altered molecular phenotype mediates increased clearance of Aβ and reduced elaboration of neurotoxic ROS.

We hypothesize that BMT results in reduced oxidative stress and mitigates neurotoxicity, possibly through MHC class II related pathways. RGCL neuron apoptosis is associated with increased production of Aβ and is reversed by inhibition of Aβ formation and aggregation [22]. Further, Aβ-induced chronic activation of glial cells results in progressive atrophy of retinal neurons *in vivo* [77] and Aβ has been shown to damage neurons by stimulating inflammation and microglia activation [78], [79]. Finally, activated microglia cells express neurotoxic cytokines and small reactive molecules, including ROS, which cause RGC degeneration. We suggest a pathogenic mechanism in which age-related neurotoxicity [80] is exacerbated by Aβ peptide deposition, and MHC class II expressing BMT-derived microglia suppress this response (Fig. 9). Studies to further elucidate differences between endogenous and donor-derived microglia will be critical to developing future microglia based therapies for neurodegenerative disease.

## References

[1] Jackson GR, Owsley C (2003) Visual dysfunction, neurodegenerative diseases, and aging. Neurol Clin 21: 709–728. 13677819 10.1016/s0733-8619(02)00107-x

[2] Lei Y, Garrahan N, Hermann B, Fautsch MP, Johnson DH, et al (2011) Transretinal degeneration in ageing human retina: a multiphoton microscopy analysis. Br J Ophthalmol 95: 727–730. 21183516 10.1136/bjo.2010.180869 PMC4406415

[3] Harwerth RS, Wheat JL, Rangaswamy NV (2008) Age-related losses of retinal ganglion cells and axons. Invest Ophthalmol Vis Sci 49: 4437–4443. 18539947 10.1167/iovs.08-1753

[4] Sung KR, Wollstein G, Bilonick RA, Townsend KA, Ishikawa H, et al (2009) Effects of age on optical coherence tomography measurements of healthy retinal nerve fiber layer, macula, and optic nerve head. Ophthalmology 116: 1119–1124. 19376593 10.1016/j.ophtha.2009.01.004 PMC2747246

[5] Curcio CA, Drucker DN (1993) Retinal ganglion cells in Alzheimer's disease and aging. Ann Neurol 33: 248–257. 8498808 10.1002/ana.410330305

[6] Balazsi AG, Rootman J, Drance SM, Schulzer M, Douglas GR (1984) The effect of age on the nerve fiber population of the human optic nerve. Am J Ophthalmol 97: 760–766. 6731540 10.1016/0002-9394(84)90509-9

[7] Loffler KU, Edward DP, Tso MO (1995) Immunoreactivity against tau, amyloid precursor protein, and beta-amyloid in the human retina. Invest Ophthalmol Vis Sci 36: 24–31. 7822152

[8] Iseri PK, Altinas O, Tokay T, Yuksel N (2006) Relationship between cognitive impairment and retinal morphological and visual functional abnormalities in Alzheimer disease. J Neuroophthalmol 26: 18–24. 16518161 10.1097/01.wno.0000204645.56873.26

[9] Blanks JC, Hinton DR, Sadun AA, Miller CA (1989) Retinal ganglion cell degeneration in Alzheimer's disease. Brain Res 501: 364–372. 2819446 10.1016/0006-8993(89)90653-7

[10] Paquet C, Boissonnot M, Roger F, Dighiero P, Gil R, et al (2007) Abnormal retinal thickness in patients with mild cognitive impairment and Alzheimer's disease. Neurosci Lett 420: 97–99. 17543991 10.1016/j.neulet.2007.02.090

[11] Danesh-Meyer HV, Birch H, Ku JY, Carroll S, Gamble G (2006) Reduction of optic nerve fibers in patients with Alzheimer disease identified by laser imaging. Neurology 67: 1852–1854. 17130422 10.1212/01.wnl.0000244490.07925.8b

[12] Sonnen JA, Larson EB, Crane PK, Haneuse S, Li G, et al (2007) Pathological correlates of dementia in a longitudinal, population-based sample of aging. Ann Neurol 62: 406–413. 17879383 10.1002/ana.21208

[13] Gomez-Isla T, Spires T, De Calignon A, Hyman BT (2008) Neuropathology of Alzheimer's disease. Handb Clin Neurol 89: 233–243. 18631748 10.1016/S0072-9752(07)01222-5

[14] Hardy J, Selkoe DJ (2002) The amyloid hypothesis of Alzheimer's disease: progress and problems on the road to therapeutics. Science 297: 353–356. 12130773 10.1126/science.1072994

[15] Yankner BA, Lu T (2009) Amyloid beta-protein toxicity and the pathogenesis of Alzheimer disease. J Biol Chem 284: 4755–4759. 18957434 10.1074/jbc.R800018200 PMC2643502

[16] Koronyo-Hamaoui M, Koronyo Y, Ljubimov AV, Miller CA, Ko MK, et al (2011) Identification of amyloid plaques in retinas from Alzheimer's patients and noninvasive in vivo optical imaging of retinal plaques in a mouse model. Neuroimage 54 Suppl 1S204–217. 20550967 10.1016/j.neuroimage.2010.06.020 PMC2991559

[17] Goldstein LE, Muffat JA, Cherny RA, Moir RD, Ericsson MH, et al (2003) Cytosolic beta-amyloid deposition and supranuclear cataracts in lenses from people with Alzheimer's disease. Lancet 361: 1258–1265. 12699953 10.1016/S0140-6736(03)12981-9

[18] Ohno-Matsui K (2011) Parallel findings in age-related macular degeneration and Alzheimer's disease. Prog Retin Eye Res 30: 217–238. 21440663 10.1016/j.preteyeres.2011.02.004

[19] Guo L, Duggan J, Cordeiro MF (2010) Alzheimer's disease and retinal neurodegeneration. Curr Alzheimer Res 7: 3–14. 20205667 10.2174/156720510790274491

[20] Johnson LV, Leitner WP, Rivest AJ, Staples MK, Radeke MJ, et al (2002) The Alzheimer's A beta -peptide is deposited at sites of complement activation in pathologic deposits associated with aging and age-related macular degeneration. Proc Natl Acad Sci U S A 99: 11830–11835. 12189211 10.1073/pnas.192203399 PMC129354

[21] Lambert MP, Barlow AK, Chromy BA, Edwards C, Freed R, et al (1998) Diffusible, nonfibrillar ligands derived from Abeta1-42 are potent central nervous system neurotoxins. Proc Natl Acad Sci U S A 95: 6448–6453. 9600986 10.1073/pnas.95.11.6448 PMC27787

[22] Perez SE, Lumayag S, Kovacs B, Mufson EJ, Xu S (2009) Beta-amyloid deposition and functional impairment in the retina of the APPswe/PS1DeltaE9 transgenic mouse model of Alzheimer's disease. Invest Ophthalmol Vis Sci 50: 793–800. 18791173 10.1167/iovs.08-2384 PMC3697019

[23] De Felice FG, Wu D, Lambert MP, Fernandez SJ, Velasco PT, et al (2008) Alzheimer's disease-type neuronal tau hyperphosphorylation induced by A beta oligomers. Neurobiol Aging 29: 1334–1347. 17403556 10.1016/j.neurobiolaging.2007.02.029 PMC3142933

[24] Heneka MT, O'Banion MK (2007) Inflammatory processes in Alzheimer's disease. J Neuroimmunol 184: 69–91. 17222916 10.1016/j.jneuroim.2006.11.017

[25] Langmann T (2007) Microglia activation in retinal degeneration. J Leukoc Biol 81: 1345–1351. 17405851 10.1189/jlb.0207114

[26] Karlstetter M, Ebert S, Langmann T (2010) Microglia in the healthy and degenerating retina: insights from novel mouse models. Immunobiology 215: 685–691. 20573418 10.1016/j.imbio.2010.05.010

[27] Frautschy SA, Yang F, Irrizarry M, Hyman B, Saido TC, et al (1998) Microglial response to amyloid plaques in APPsw transgenic mice. Am J Pathol 152: 307–317. 9422548 PMC1858113

[28] D'Andrea MR, Cole GM, Ard MD (2004) The microglial phagocytic role with specific plaque types in the Alzheimer disease brain. Neurobiol Aging 25: 675–683. 15172747 10.1016/j.neurobiolaging.2003.12.026

[29] Rogers J, Strohmeyer R, Kovelowski CJ, Li R (2002) Microglia and inflammatory mechanisms in the clearance of amyloid beta peptide. Glia 40: 260–269. 12379913 10.1002/glia.10153

[30] Agostinho P, Cunha RA, Oliveira C (2010) Neuroinflammation, oxidative stress and the pathogenesis of Alzheimer's disease. Curr Pharm Des 16: 2766–2778. 20698820 10.2174/138161210793176572

[31] Prinz M, Mildner A (2011) Microglia in the CNS: immigrants from another world. Glia 59: 177–187. 21125659 10.1002/glia.21104

[32] Keene CD, Chang RC, Lopez-Yglesias AH, Shalloway BR, Sokal I, et al (2010) Suppressed accumulation of cerebral amyloid {beta} peptides in aged transgenic Alzheimer's disease mice by transplantation with wild-type or prostaglandin E2 receptor subtype 2-null bone marrow. Am J Pathol 177: 346–354. 20522650 10.2353/ajpath.2010.090840 PMC2893677

[33] Simard AR, Soulet D, Gowing G, Julien JP, Rivest S (2006) Bone marrow-derived microglia play a critical role in restricting senile plaque formation in Alzheimer's disease. Neuron 49: 489–502. 16476660 10.1016/j.neuron.2006.01.022

[34] Malm TM, Koistinaho M, Parepalo M, Vatanen T, Ooka A, et al (2005) Bone-marrow-derived cells contribute to the recruitment of microglial cells in response to beta-amyloid deposition in APP/PS1 double transgenic Alzheimer mice. Neurobiol Dis 18: 134–142. 15649704 10.1016/j.nbd.2004.09.009

[35] Priller J, Flugel A, Wehner T, Boentert M, Haas CA, et al (2001) Targeting gene-modified hematopoietic cells to the central nervous system: use of green fluorescent protein uncovers microglial engraftment. Nat Med 7: 1356–1361. 11726978 10.1038/nm1201-1356

[36] Kaneko H, Nishiguchi KM, Nakamura M, Kachi S, Terasaki H (2008) Characteristics of bone marrow-derived microglia in the normal and injured retina. Invest Ophthalmol Vis Sci 49: 4162–4168. 18487364 10.1167/iovs.08-1738

[37] Xu H, Chen M, Mayer EJ, Forrester JV, Dick AD (2007) Turnover of resident retinal microglia in the normal adult mouse. Glia 55: 1189–1198. 17600341 10.1002/glia.20535

[38] Holcomb L, Gordon MN, McGowan E, Yu X, Benkovic S, et al (1998) Accelerated Alzheimer-type phenotype in transgenic mice carrying both mutant amyloid precursor protein and presenilin 1 transgenes. Nat Med 4: 97–100. 9427614 10.1038/nm0198-097

[39] Jankowsky JL, Slunt HH, Ratovitski T, Jenkins NA, Copeland NG, et al (2001) Co-expression of multiple transgenes in mouse CNS: a comparison of strategies. Biomol Eng 17: 157–165. 11337275 10.1016/s1389-0344(01)00067-3

[40] Borchelt DR, Ratovitski T, van Lare J, Lee MK, Gonzales V, et al (1997) Accelerated amyloid deposition in the brains of transgenic mice coexpressing mutant presenilin 1 and amyloid precursor proteins. Neuron 19: 939–945. 9354339 10.1016/s0896-6273(00)80974-5

[41] Gough PJ, Gomez IG, Wille PT, Raines EW (2006) Macrophage expression of active MMP-9 induces acute plaque disruption in apoE-deficient mice. J Clin Invest 116: 59–69. 16374516 10.1172/JCI25074 PMC1319218

[42] Quinn JF, Bussiere JR, Hammond RS, Montine TJ, Henson E, et al (2007) Chronic dietary alpha-lipoic acid reduces deficits in hippocampal memory of aged Tg2576 mice. Neurobiol Aging 28: 213–225. 16448723 10.1016/j.neurobiolaging.2005.12.014

[43] Boettcher C, Ulbricht E, Helmlinger D, Mack AF, Reichenbach A, et al (2008) Long-term engraftment of systemically transplanted, gene-modified bone marrow-derived cells in the adult mouse retina. Br J Ophthalmol 92: 272–275. 18227206 10.1136/bjo.2007.126318

[44] Leinonen V, Koivisto AM, Savolainen S, Rummukainen J, Tamminen JN, et al (2010) Amyloid and tau proteins in cortical brain biopsy and Alzheimer's disease. Ann Neurol 68: 446–453. 20976765 10.1002/ana.22100

[45] Gotz J, Chen F, van Dorpe J, Nitsch RM (2001) Formation of neurofibrillary tangles in P301l tau transgenic mice induced by Abeta 42 fibrils. Science 293: 1491–1495. 11520988 10.1126/science.1062097

[46] Alonso AC, Grundke-Iqbal I, Iqbal K (1996) Alzheimer's disease hyperphosphorylated tau sequesters normal tau into tangles of filaments and disassembles microtubules. Nat Med 2: 783–787. 8673924 10.1038/nm0796-783

[47] Loo DT, Copani A, Pike CJ, Whittemore ER, Walencewicz AJ, et al (1993) Apoptosis is induced by beta-amyloid in cultured central nervous system neurons. Proc Natl Acad Sci U S A 90: 7951–7955. 8367446 10.1073/pnas.90.17.7951 PMC47265

[48] Nakagawa T, Zhu H, Morishima N, Li E, Xu J, et al (2000) Caspase-12 mediates endoplasmic-reticulum-specific apoptosis and cytotoxicity by amyloid-beta. Nature 403: 98–103. 10638761 10.1038/47513

[49] Miller NR (1990) Alzheimer's disease, optic neuropathy, and selective ganglion cell damage. Ophthalmology 97: 7–8. 2314846

[50] Tetewsky SJ, Duffy CJ (1999) Visual loss and getting lost in Alzheimer's disease. Neurology 52: 958–965. 10102412 10.1212/wnl.52.5.958

[51] Keri S, Antal A, Kalman J, Janka Z, Benedek G (1999) Early visual impairment is independent of the visuocognitive and memory disturbances in Alzheimer's disease. Vision Res 39: 2261–2265. 10343807 10.1016/s0042-6989(98)00310-1

[52] Parisi V, Restuccia R, Fattapposta F, Mina C, Bucci MG, et al (2001) Morphological and functional retinal impairment in Alzheimer's disease patients. Clin Neurophysiol 112: 1860–1867. 11595144 10.1016/s1388-2457(01)00620-4

[53] Howell GR, Soto I, Zhu X, Ryan M, Macalinao DG, et al (2012) Radiation treatment inhibits monocyte entry into the optic nerve head and prevents neuronal damage in a mouse model of glaucoma. J Clin Invest 122: 1246–1261. 22426214 10.1172/JCI61135 PMC3314470

[54] Bosco A, Crish SD, Steele MR, Romero CO, Inman DM, et al (2012) Early reduction of microglia activation by irradiation in a model of chronic glaucoma. PLoS One 7: e43602. 22952717 10.1371/journal.pone.0043602 PMC3431380

[55] Otani A, Kojima H, Guo C, Oishi A, Yoshimura N (2012) Low-dose-rate, low-dose irradiation delays neurodegeneration in a model of retinitis pigmentosa. Am J Pathol 180: 328–336. 22074737 10.1016/j.ajpath.2011.09.025

[56] Igarashi T, Miyake K, Hayakawa J, Kawabata K, Ishizaki M, et al (2007) Apoptotic cell death and regeneration in the newborn retina after irradiation prior to bone marrow transplantation. Curr Eye Res 32: 543–553. 17612970 10.1080/02713680701389333

[57] Herzog KH, Schulz A, Buerkle C, Gromoll C, Braun JS (2007) Radiation-induced apoptosis in retinal progenitor cells is p53-dependent with caspase-independent DNA fragmentation. Eur J Neurosci 25: 1349–1356. 17425561 10.1111/j.1460-9568.2007.05381.x

[58] Borges HL, Chao C, Xu Y, Linden R, Wang JY (2004) Radiation-induced apoptosis in developing mouse retina exhibits dose-dependent requirement for ATM phosphorylation of p53. Cell Death Differ 11: 494–502. 14752509 10.1038/sj.cdd.4401366

[59] Schmidt SL, Vitral RW, Linden R (2001) Effects of prenatal ionizing irradiation on the development of the ganglion cell layer of the mouse retina. Int J Dev Neurosci 19: 469–473. 11378306 10.1016/s0736-5748(00)00068-x

[60] Chen L, Yang P, Kijlstra A (2002) Distribution, markers, and functions of retinal microglia. Ocul Immunol Inflamm 10: 27–39. 12461701 10.1076/ocii.10.1.27.10328

[61] Dringen R (2005) Oxidative and antioxidative potential of brain microglial cells. Antioxid Redox Signal 7: 1223–1233. 16115027 10.1089/ars.2005.7.1223

[62] Wang AL, Lukas TJ, Yuan M, Neufeld AH (2010) Age-related increase in mitochondrial DNA damage and loss of DNA repair capacity in the neural retina. Neurobiol Aging 31: 2002–2010. 19084291 10.1016/j.neurobiolaging.2008.10.019

[63] Eglitis MA, Mezey E (1997) Hematopoietic cells differentiate into both microglia and macroglia in the brains of adult mice. Proc Natl Acad Sci U S A 94: 4080–4085. 9108108 10.1073/pnas.94.8.4080 PMC20571

[64] Simard AR, Rivest S (2004) Bone marrow stem cells have the ability to populate the entire central nervous system into fully differentiated parenchymal microglia. Faseb J 18: 998–1000. 15084516 10.1096/fj.04-1517fje

[65] Prinz M, Priller J, Sisodia SS, Ransohoff RM (2011) Heterogeneity of CNS myeloid cells and their roles in neurodegeneration. Nat Neurosci 14: 1227–1235. 21952260 10.1038/nn.2923

[66] Mizutani M, Pino PA, Saederup N, Charo IF, Ransohoff RM, et al (2012) The fractalkine receptor but not CCR2 is present on microglia from embryonic development throughout adulthood. J Immunol 188: 29–36. 22079990 10.4049/jimmunol.1100421 PMC3244524

[67] Kennedy DW, Abkowitz JL (1997) Kinetics of central nervous system microglial and macrophage engraftment: analysis using a transgenic bone marrow transplantation model. Blood 90: 986–993. 9242527

[68] Anderson MG, Libby RT, Gould DB, Smith RS, John SW (2005) High-dose radiation with bone marrow transfer prevents neurodegeneration in an inherited glaucoma. Proc Natl Acad Sci U S A 102: 4566–4571. 15758074 10.1073/pnas.0407357102 PMC555465

[69] Rogers J, Lue LF (2001) Microglial chemotaxis, activation, and phagocytosis of amyloid beta-peptide as linked phenomena in Alzheimer's disease. Neurochem Int 39: 333–340. 11578768 10.1016/s0197-0186(01)00040-7

[70] Mildner A, Schmidt H, Nitsche M, Merkler D, Hanisch UK, et al (2007) Microglia in the adult brain arise from Ly-6ChiCCR2+ monocytes only under defined host conditions. Nat Neurosci 10: 1544–1553. 18026096 10.1038/nn2015

[71] Yin Y, Cui Q, Li Y, Irwin N, Fischer D, et al (2003) Macrophage-derived factors stimulate optic nerve regeneration. J Neurosci 23: 2284–2293. 12657687 10.1523/JNEUROSCI.23-06-02284.2003 PMC6742044

[72] Otth C, Concha II, Arendt T, Stieler J, Schliebs R, et al (2002) AbetaPP induces cdk5-dependent tau hyperphosphorylation in transgenic mice Tg2576. J Alzheimers Dis 4: 417–430. 12446973 10.3233/jad-2002-4508

[73] Roberson ED, Scearce-Levie K, Palop JJ, Yan F, Cheng IH, et al (2007) Reducing endogenous tau ameliorates amyloid beta-induced deficits in an Alzheimer's disease mouse model. Science 316: 750–754. 17478722 10.1126/science.1141736

[74] Gupta N, Fong J, Ang LC, Yucel YH (2008) Retinal tau pathology in human glaucomas. Can J Ophthalmol 43: 53–60. 18219347 10.3129/i07-185

[75] Liu B, Rasool S, Yang Z, Glabe CG, Schreiber SS, et al (2009) Amyloid-peptide vaccinations reduce {beta}-amyloid plaques but exacerbate vascular deposition and inflammation in the retina of Alzheimer's transgenic mice. Am J Pathol 175: 2099–2110. 19834067 10.2353/ajpath.2009.090159 PMC2774073

[76] Blanks JC, Schmidt SY, Torigoe Y, Porrello KV, Hinton DR, et al.. (1996) Retinal pathology in Alzheimer's disease. II. Regional neuron loss and glial changes in GCL. Neurobiol Aging. 385–395. 10.1016/0197-4580(96)00009-7 8725900

[77] Walsh DT, Bresciani L, Saunders D, Manca MF, Jen A, et al (2005) Amyloid beta peptide causes chronic glial cell activation and neuro-degeneration after intravitreal injection. Neuropathol Appl Neurobiol 31: 491–502. 16150120 10.1111/j.1365-2990.2005.00666.x

[78] Golde TE (2003) Alzheimer disease therapy: can the amyloid cascade be halted? J Clin Invest 111: 11–18. 12511580 10.1172/JCI17527 PMC151845

[79] Wada R, Tifft CJ, Proia RL (2000) Microglial activation precedes acute neurodegeneration in Sandhoff disease and is suppressed by bone marrow transplantation. Proc Natl Acad Sci U S A 97: 10954–10959. 11005868 10.1073/pnas.97.20.10954 PMC27130

[80] Minghetti L, Levi G (1998) Microglia as effector cells in brain damage and repair: focus on prostanoids and nitric oxide. Prog Neurobiol 54: 99–125. 9460796 10.1016/s0301-0082(97)00052-x

